# Non-invasive nanosecond electroporation for biocontrol of surface infections: an *in vivo* study

**DOI:** 10.1038/s41598-018-32783-7

**Published:** 2018-09-28

**Authors:** Vitalij Novickij, Auksė Zinkevičienė, Emilija Perminaitė, Robertas Čėsna, Eglė Lastauskienė, Algimantas Paškevičius, Jurgita Švedienė, Svetlana Markovskaja, Jurij Novickij, Irutė Girkontaitė

**Affiliations:** 1 Institute of High Magnetic Fields, Vilnius Gediminas Technical University, Vilnius, Lithuania; 2 State Research Institute Centre for Innovative Medicine, Department of Immunology, Vilnius, Lithuania; 3 Institute of Biosciences, Life Sciences Centre, Vilnius University, Vilnius, Lithuania; 4 Laboratory of Biodeterioration Research, Nature Research Centre, Vilnius, Lithuania; 5 Laboratory of Mycology, Nature Research Centre, Vilnius, Lithuania

## Abstract

Invasive infections caused by drug-resistant bacteria are frequently responsible for fatal sepsis, morbidity and mortality rates. In this work, we propose a new methodology based on nanosecond high frequency electric field bursts, which enables successful eradication of bacteria *in vivo*. High frequency (15 kHz) 15–25 kV/cm 300–900 ns pulsing bursts were used separately and in combination with acetic acid (0.1**–**1%) to treat *Pseudomonas aeruginosa* in a murine model. Acetic acid 1% alone was effective resulting in almost 10-fold reduction of bacteria viability, however combination of nanosecond electric field and acetic acid 1% treatment was the most successful showing almost full eradication (0.01% survival compared to control) of the bacteria in the contaminated area. The short duration of the pulses (sub-microsecond) and high frequency (kHz range) of the burst enabled reduction of the muscle contractions to barely detectable level while the proposed applicators ensured predominantly topical treatment, without electroporation of deeper tissues. The results of our study have direct application for treatment of wounds and ulcers when chemical treatment is no longer effective.

## Introduction

Invasive infections caused by drug-resistant bacteria are becoming increasingly prevalent and are associated with high morbidity and mortality rates, especially in patients with burn wounds^1–4^. The management of an infection in thermal injury presents challenges in terms of clinical diagnosis and rapid definition of effective antimicrobial therapy^4,5^. Topical antibiotics are effective in reducing such wound infections, however the absolute and long-term benefit is small^6^ since the accelerated evolution of bacterial resistance is frequently associated with a widespread use of antibiotics^7,8^. Therefore, with the emergence of multidrug-resistant strains, alternative strategies to control or prevent infections must be developed in parallel with novel antimicrobial agents^9–11^.

Novel chemical antimicrobial compounds (antimicrobial peptides, bioactive nanoparticles, etc.) are usually associated with the specific mechanism of biointeraction^12–14^. As a result, the microbial inactivation efficacy varies dependent on the biological object, while the high resistance is common in gram-negative bacteria^15–17^. One of the solutions is the application of alternative universal agents, such as acetic, ascorbic or other acids for pH manipulation and sterilization of the wound^18,19^. Acetic acid (AA) already proved to be efficient against bacteria biofilms and treatment of ulcers, however physiologically tolerable concentrations should be used^20^. To prevent corrosive effects of acid the concentration should be <10%, while up to 5% are considered harmless^20–22^. Since a trade-off between the treatment efficiency and patient-friendly procedure is required, the research and application of additional physical methods in combination with acidic treatment is performed^23^. In this work, we propose nanosecond range electroporation as an effective tool for biocontrol of surface infections.

Electroporation is a pulsed electric field (PEF) induced phenomenon of increased cell membrane permeability^24,25^, which has found a variety of applications in food processing industry (bacterial decontamination)^26^, biotechnology (protein extraction, transformation)^27–29^ and cancer treatment (tissue ablation, electrochemotherapy)^30,31^. It is an electric pulse-dependent methodology, thus both the positive and side effects of the treatment depend on the parameters of electric field and the structure of electrodes (applicators)^32,33^. In addition, bacteria are less susceptible to electroporation compared to mammalian cells (due to cell size, cell wall, internal structure)^34^. Therefore, the parameters of PEF that are required to inactivate the bacteria or pathogenic yeasts^35–37^ will likely to result in tissue ablation of the treated area, which is non-desirable and thus it was believed that electroporation is not suitable for wound sterilization. Nevertheless, recently Golberg *et al*. showed a concept of electroporation-assisted wound sterilization by means of microsecond electroporation^38^. The group used 5 kV/cm × 70 µs × 80 pulses delivered at repetition frequency of 1 Hz between parallel plate electrodes, which resulted in a successful treatment. However, the methodology still has considerable problems requiring solution: (1) positioning and the type of used electrodes significantly limit the application only to topical skin infections; (2) the skin is damaged by electroporation due to long and high amplitude PEF pulses; (3) the treatment is relatively long, which limits the possibilities of coverage of large surface area; (4) muscle contractions are severe when PEF of such intensity and duration is used.

Therefore, in this work we propose a new methodology based on nanosecond high frequency PEF bursts, which provides solution to all of the described problems. As a bacterial model, the gram-negative *Pseudomonas aeruginosa* was used.

*Pseudomonas aeruginosa* is one of the most frequently responsible bacteria for fatal sepsis in burn wound patients and is in the top of the list of the drug resistive bacteria, which have the highest threat to human health according to World Health Organization^1,39,40^. As a result, we present a new method and *in vitro* and *in vivo* data on successful eradication of this bacteria using nanosecond range electroporation separately and in combination with acetic acid.

## Results

### Proposed methodology

**Figure 1:**
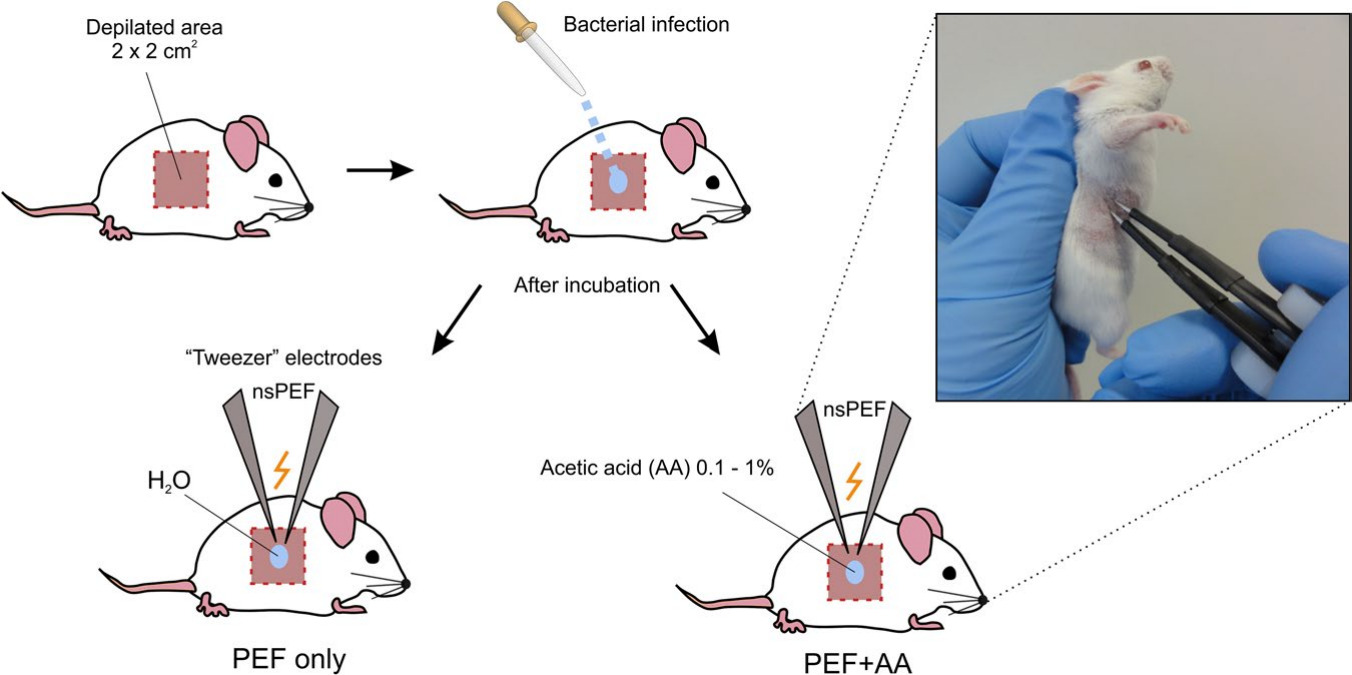
The schematic representation of proposed electroporation mediated methodology for biocontrol of surface infections, where PEF – pulsed electric field; nsPEF – high frequency nanosecond PEF bursts. Distilled water or low concentrations of acetic acid were used as an electrode-skin interface.

In this work, we propose a new methodology based on nanosecond high frequency PEF bursts for treatment of surface infections. The schematic of the proposed methodology is shown in Fig. 1.

The basepoint is the short duration (nanosecond) of the electric field pulses (nsPEF), which combined with the high frequency (kHz range) of the burst allows to reduce the muscle contractions compared to available procedures. The “tweezer” electrodes and the topical delivery of low conductive liquid as an electrode-skin interface allows to ensure predominantly topical treatment, without electroporation of deeper tissues. The application of acetic acid enables additive and a more effective procedure, while the treatment being fast and flexible with capability to ensure a safety margin in pulse energy and/or acid concentration.

### *In vitro* model

**Figure 2:**
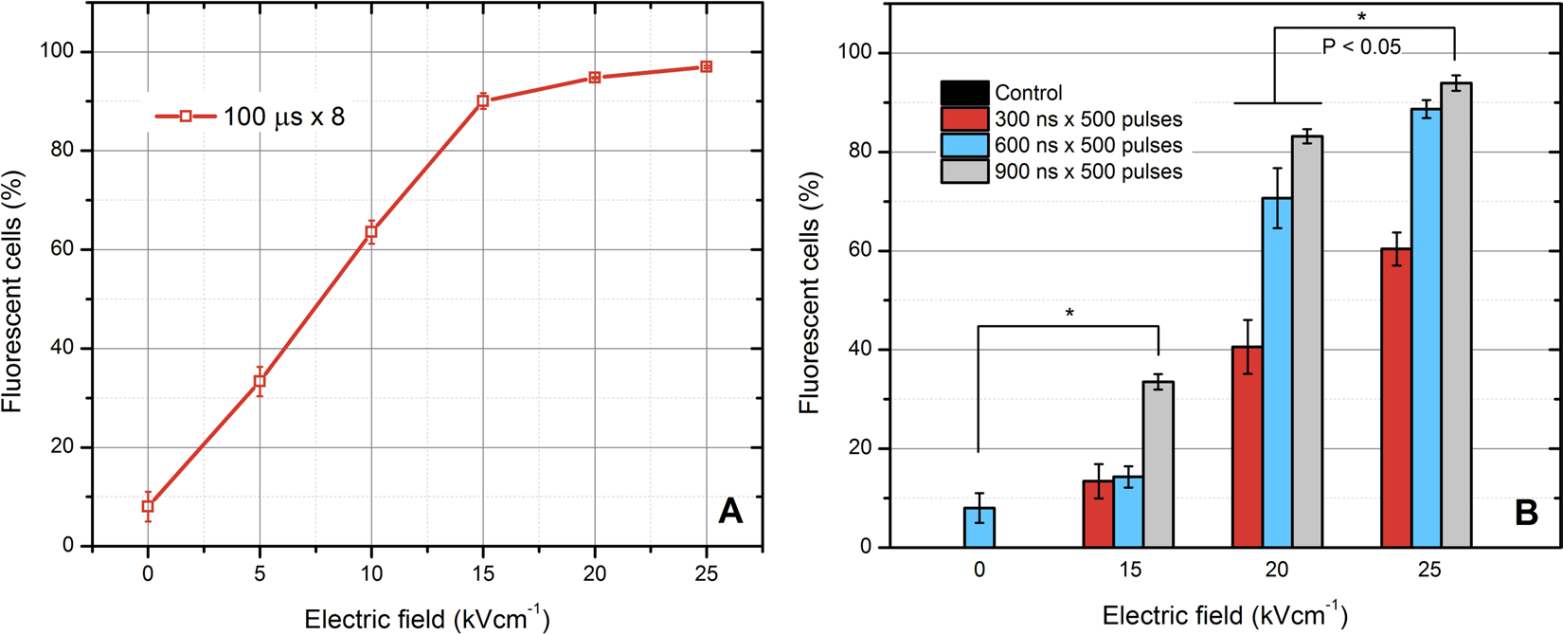
The dependence of *P*. *aeruginosa* permeabilization on the applied pulsed electric field parameters, where (**A**) microsecond range protocols; (**B**) nanosecond range protocols. The saturated permeabilization is achieved when the electric field amplitude exceeds 20 kV/cm. The 25 kV/cm, 900 ns protocol was defined as optimal due to high permeabilization rate of bacteria.

For selection of optimal parameters for the *in vivo* model, firstly we analyzed the dependence of bacteria permeabilization on PEF parameters *in vitro*. Bursts of nanosecond (300–900 ns) pulses of varied intensity (15–25 kV/cm) were delivered and the uptake of PI was evaluated using flow cytometry. Conventional microsecond electroporation (100 µs × 8) was used a reference. The results are summarized in Fig. 2.

As it can be seen in Fig. 2A the *P*. *aeruginosa* requires PEF above 15 kV/cm to achieve high permeabilization rate. The result can be improved by increase of the number of pulses or pulse duration, however it is no applicable in the microsecond range due to possible severe muscle contractions. Also, the amplitude is already 10-fold higher compared to PEF, which is required to achieve high permeabilization in mammalian cells^41^.

Similar tendency was observed when bursts of nanosecond pulses were used (Fig. 2B). The saturated permeabilization was achieved only when the 25 kV/cm × 900 ns × 500 pulses protocol (P1) was applied. The permeabilization rate varied in a dose dependent manner. Therefore, the P1 protocol was selected as optimal (due to saturated permeabilization) and the number of pulses was doubled (25 kV/cm × 900 ns × 1000 – protocol P2) for further study to induce significant irreversible electroporation for inactivation of *P*. *aeruginosa*.

**Figure 3:**
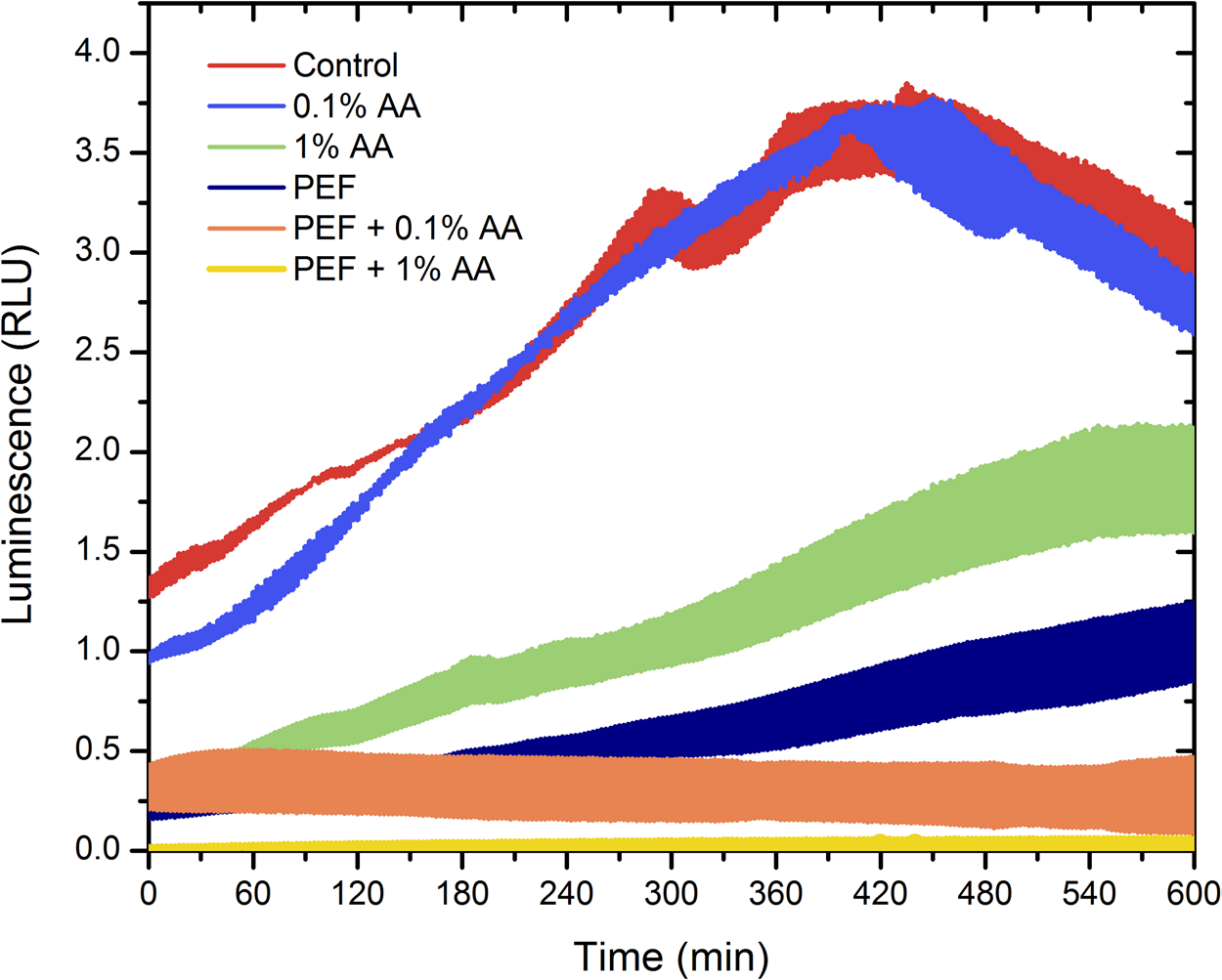
The dependence of *P*. *aeruginosa* luminescence on the applied treatment parameters, live bacteria luminesce; the width of colored area corresponds to standard deviation of data during selected treatment protocol; PEF – 25 kV/cm × 900 ns × 1000 pulses; AA – acetic acid. The 1% acetic acid combined with electroporation allowed complete inactivation of bacteria *in vitro*, which is unachievable if the treatments are used separately.

The inhibition efficacy of the P2 protocol was evaluated dynamically every 2.5 min for 10 h by measurement of the luminescence of the *P*. *aeruginosa*. The treatment was accompanied by addition of 0.1% and 1% AA during electroporation for determination of an additive effect. The results are summarized in Fig. 3.

As it can be seen in Fig. 3. PEF itself (P2 protocol) reduced the luminescence of bacteria more than 5 times, however it was not sufficient to prevent further growth of *P*. *aeruginosa* and after 10 h the luminescence was comparable to the beginning of the experiment. Similar effect was observed when the 1% AA was used. The 0.1% AA had no effect on the luminescence of the *P*. *aeruginosa* due to insufficient concentration. However, in all experimental instances where combination of two procedures was used an additive effect (PEF + AA) was observed. The 1% acetic acid combined with electroporation allowed complete inactivation of bacteria *in vitro*, which was unachievable if the treatments were used separately. The width of colored area corresponds to standard deviation of data between independent experimental repetitions.

### *In vivo* model

**Figure 4:**
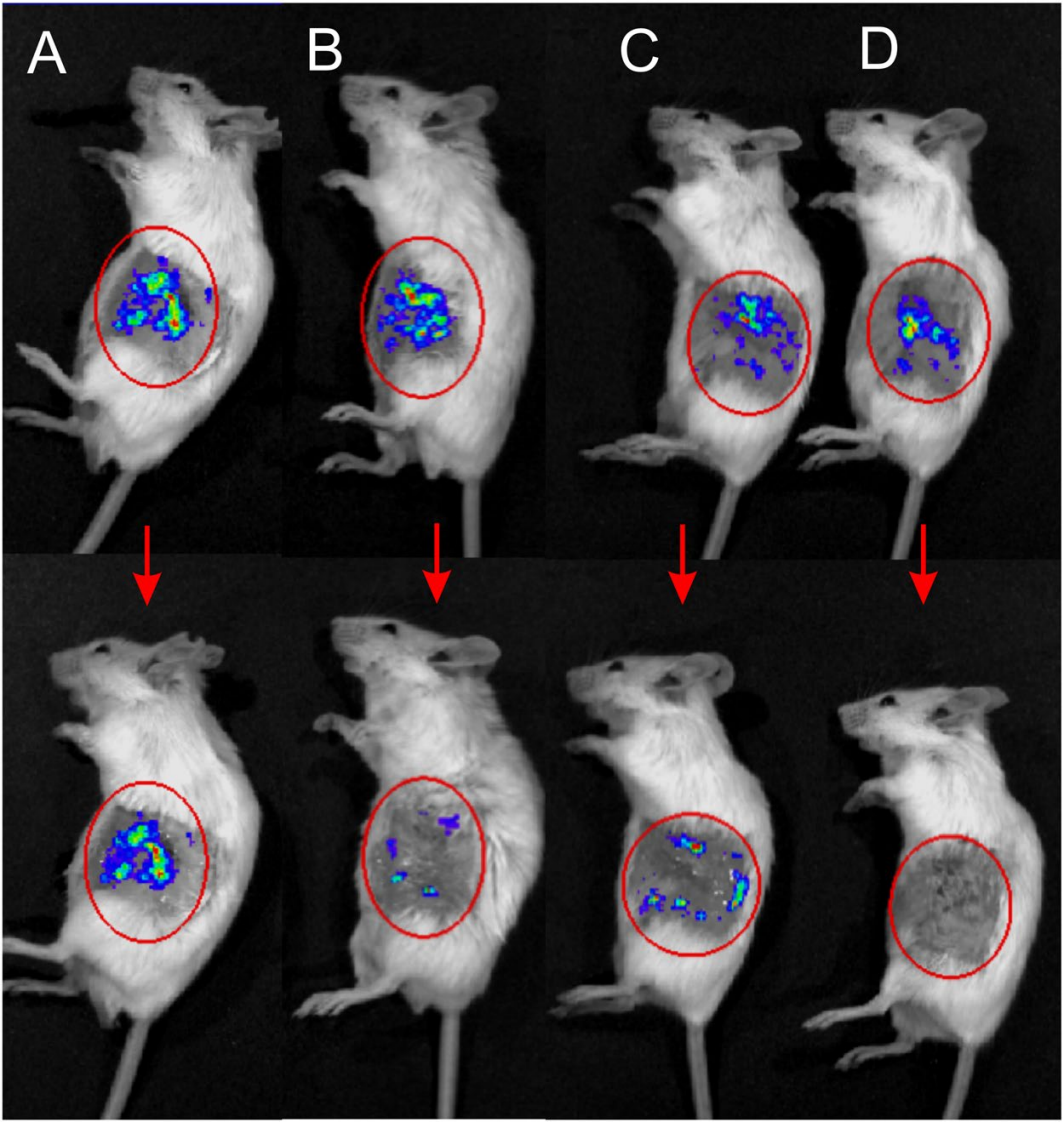
The mice before treatment on the top and the same mice after the treatment on the bottom, where (**A**) treated with distilled water; (**B**) treated with distilled water and electroporation (25 kV/cm × 900 ns × 1000 pulses); (**C**) treated with acetic acid 1%; (**D**) treated with acetic acid 1% and electroporation. Combined treatment allows full eradication of bacteria.

Based on the positive *in vitro* results, the methodology was tested *in vivo*. The exemplary images adequately representing the treatment efficiency using different protocols are shown in Fig. 4.

**Figure 5:**
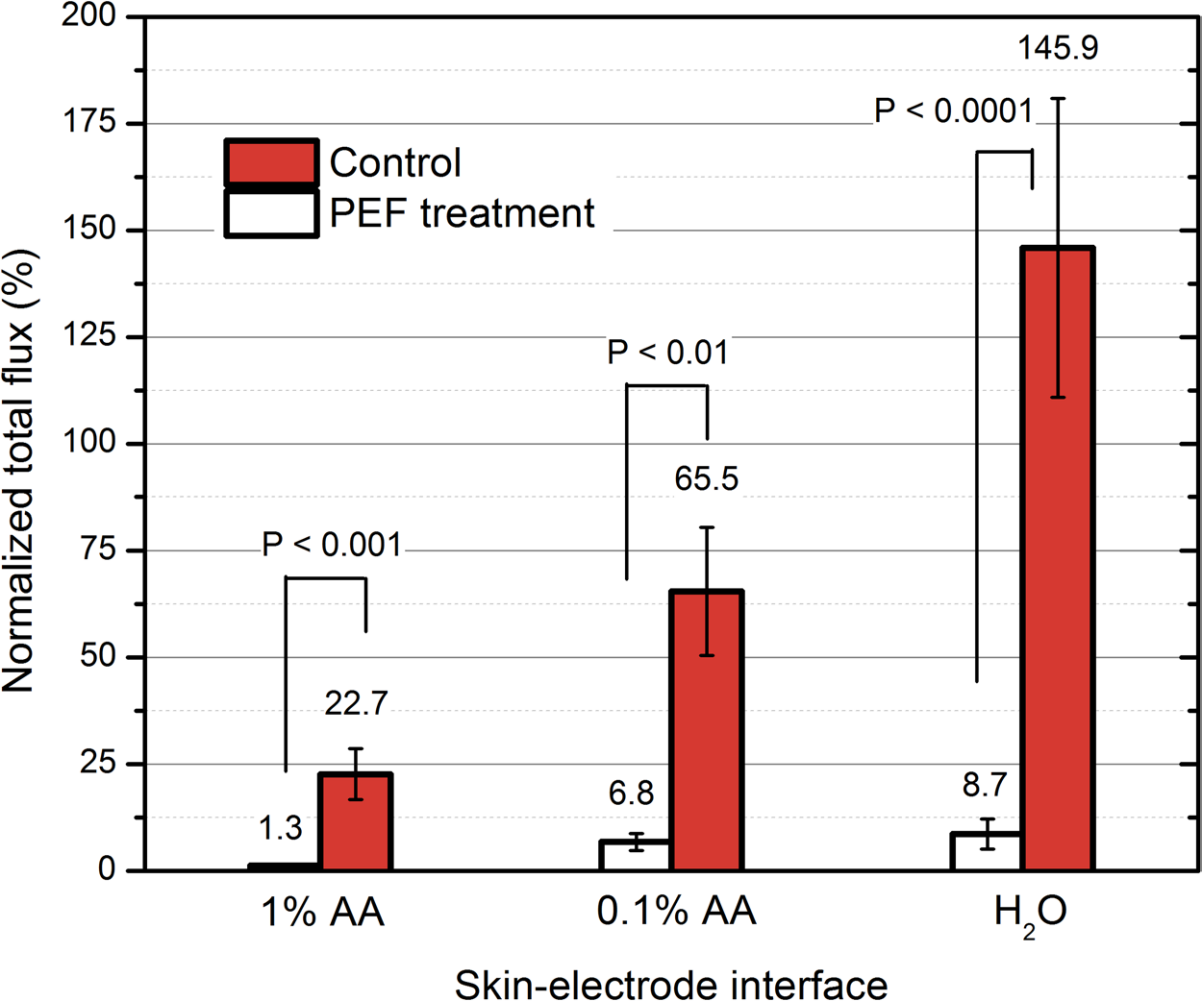
The *in vivo* potential of acetic acid and electroporation for inactivation of *P*. *aeruginosa*. The combined treatment (AA 1% and PEF) results in up to full eradication of bacteria, where PEF: 25 kV/cm × 900 ns × 1000 pulses delivered at pulse repetition frequency of 15 kHz.

Both the acetic acid and electroporation treatment reduced the viability of bacteria. The quantified results are summarized in Fig. 5.

The luminescence (representing the extent of bacterial contamination) increased up to 46 ± 34% when the contaminated area was treated solely by distilled water. Acetic acid 1% alone was effective resulting in almost 10-fold reduction of *P*. *aeruginosa* luminescence, however we were also able to observe inhibition of *P*. *aeruginosa* when 0.1% AA was used, which was not the case *in vitro*. Both concentrations of acetic acid were used *in vivo* to highlight the fact that if the concentration of acetic acid is too low (i.e. 0.1%), fraction of bacteria will remain viable and potentially the infection can develop further.

The combination of PEF and acetic acid 1% treatment was the most successful resulting in almost full eradication (0.01% survival compared to control) of the bacteria in the contaminated area. However, the 0.1% AA + PEF treatment showed no statistically significant difference if compared to PEF only procedure. In all cases when PEF was used the muscle contractions were minimal due to high frequency and short duration of the burst.

## Discussion

In this work, we have demonstrated that the application of nanosecond range PEF allows countering multiple limitations of micro-millisecond protocols and enables use of electroporation for biocontrol of surface infections. We have also used acetic acid (0.1, 1%), which showed a significant additive effect with PEF methodology.

Bacteria cell wall serves as an ultimate barrier against the environment, while application of PEF triggers permeabilization of plasma membrane and disruption of the cell wall integrity^27,34^. Therefore, possible mechanism of the effect presumably lies within the physical damage that is caused by PEF. The increased efficacy of the treatment with acetic acid can be attributed to the internal pH shock and high permeabilization of bacteria, which results in uptake of acid^21,42^. Also, generation of reactive oxygen species (ROS) is frequently associated with antimicrobial efficiency^43^. In mammalian cells, it is known that nanosecond range PEF itself can generate both extracellular (electrochemical) and intracellular ROS^44^ mediating lipid peroxidation. The oxidized bilayers are leaky and prone to spontaneous pore formation, which enables enhanced passage of ions and molecules across the cell membrane^45,46^. ROS can be also harmful for cell metabolism inducing oxidative stress, starting processes of apoptosis and mutating DNA and RNA^47^. All of these factors can contribute to the increased inhibition efficacy and PEF damage. However, taking into account that without electroporation it is the acetic acid molecule itself that kills bacteria (external pH is not that significant for cell survival)^20^, currently it is not possible to define the exact additive mechanism of AA with PEF against bacteria.

We have improved the currently available PEF wound sterilization concept^38^ significantly. The short duration of the pulses (sub-microsecond) and high frequency (kHz range) of the burst allowed reducing the muscle contractions to barely detectable level. The biggest concern, was the damage of healthy tissues, which may be occurring due extremely high PEF amplitudes. Nevertheless, the solution to use “tweezer” type electrodes with liquid skin/electrode interface ensured a predominantly topical procedure. After the treatment, we were able to observe PEF induced damage of epidermis, however the skin fully regenerated in less than one week. The treatment itself is fast (less than a second), while it is limited by technological platform that is available. Our facilities allowed to apply the burst to an effective area of 5–6 mm^2^, therefore coverage of 2 × 2 cm^2^ was a matter of several minutes. It is possible to improve the parameters by developing new electroporation systems and electrodes, which cover higher effective area.

Our proposed methodology should be also applicable against pathogenic yeast, fungi and gram-positive bacteria since they are more susceptible to electroporation^47–50^. The structure of gram-negative bacteria involves a two-layer cell wall and an outer membrane, which constitutes a higher challenge for successful permeabilization using PEF compared to other biological objects, which are associated with wound infections^51,52^. It implies, that the PEF amplitude and/or number and duration of the pulses can be reduced if used on other biological objects. Lastly, electroporation is a physical method, which is non-toxic and non-dependent on drug resistance, thus it makes the wound sterilization procedure universal and applicable without diagnostics of the pathogen causing the infection. It is particularly useful during intensive-care events when time and successful management of the wound are vital^4,5^.

Future works should involve optimization of the treatment parameters and determination of the optimal AA concentration and PEF parameters to ensure painless, fast and efficient treatment.

## Material and Methods

### Pulsed power setups

The 0–3 kV, 100 ns–1 ms square wave high voltage pulse generator was used for electroporation^53^. The setup generated pulsed electric field (0–25 kV/cm) using two applicators: (1) a commercially available 1 mm gap electroporation cuvette (Biorad, Hercules, USA) for *in vitro* experiments and (2) the “tweezer” type steel electrodes with 1.2 mm gap specifically developed for *in vivo* experiments (Refer to Fig. 1).

High frequency (15 kHz) 15–25 kV/cm 300–900 ns pulsing bursts were used. We have selected durations, which are short enough to minimize muscle contractions, however above the threshold when polarization time is insufficient to cause electroporation at selected PEF intensities. For *in vitro* experiments, the number of pulses was limited to 500, while a sequence of 1000 pulses was applied *in vivo*. Conventional 100 μs × 8 pulsing protocols (0–25 kV/cm) were used as a reference *in vitro*.

**Figure 6:**
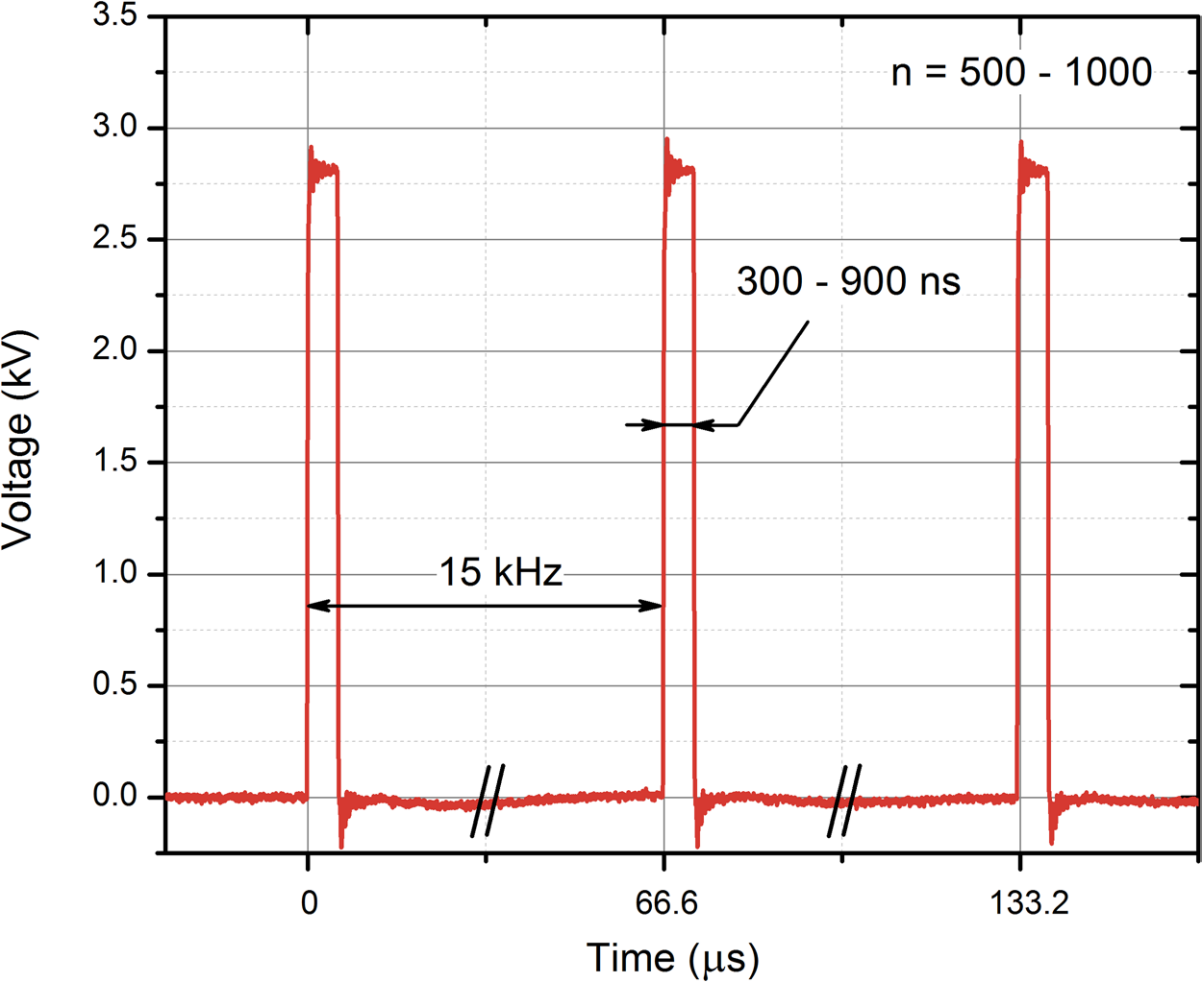
The waveform of the applied electric pulses. The pulses have been measured using a DPO4034 oscilloscope (Tektronix, Beaverton, OR, United States), post-processed in OriginPro Software (OriginLab, Northampton, MA, United States); n – number of pulses.

The waveform of the applied nanosecond pulses is shown in Fig. 6. The transient processes did not exceed more than 10% of the pulse amplitude, therefore the influence of transient processes was considered as negligible.

### Bacterial strain

*P*. *aeruginosa* ATCC27853 bacterial strain was transformed with plasmid pAKlux2 (a kind gift from Attila Karsi, Addgene plasmid #14080)^54^. This plasmid contains the luxCDABE operon made from luciferase (luxAB) and fatty acid reductase (lux CDE) genes. Therefore the live (but not dead) bacteria are bioluminescent. *P*. *aeruginosa* cells were grown overnight in liquid LB (Luria-Bertani) medium (10 g/l tryptone, 5 g/l yeast extract, 5 g/l NaCl) in the rotary shaker at 37 °C. 1 ml of the OD = 1 (600 nm; 1.5 × 10^9^ cells) cell culture was transferred to 10 ml of the fresh LB medium and grown for 4 hours at the same conditions. Later the cells were washed 3 times with 1 M sorbitol and re-suspended in 1 M sorbitol at final concentration of 10^9^.

### Permeabilization assay

**Figure 7:**
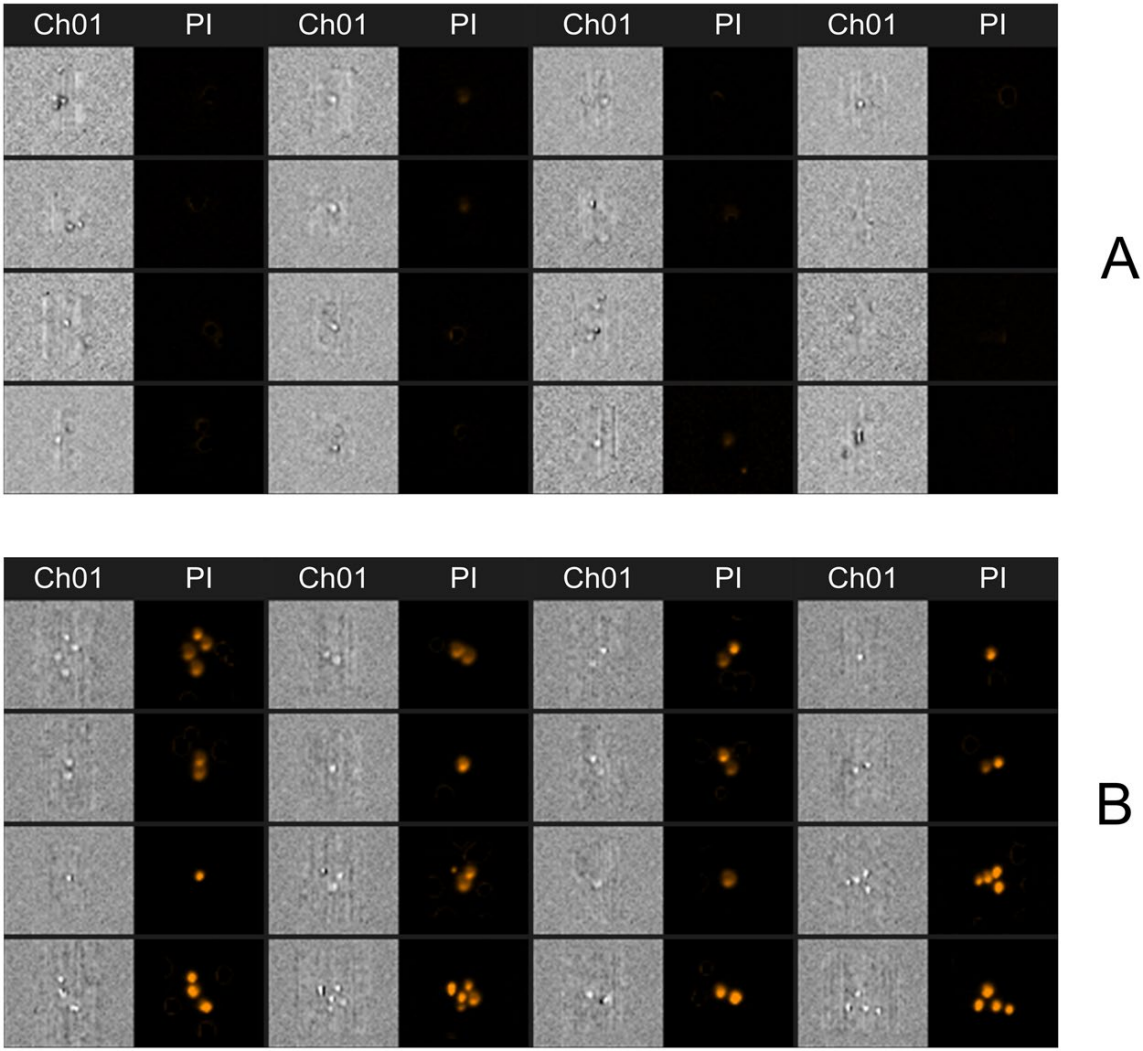
The example of fluorescence photographs of propidium iodide negative (**A**) and propidium iodide positive (**B**) cells. For better perception and indication of permeabilization multiple cells are shown, however for quantitative analysis of cell permeabilization only single cells were used. Due to electroporation (**B**) the propidium iodide can successfully enter the cells, which is not the case in the untreated control (**A**). Ch01 – brightfield image; PI – fluorescence image (488 nm), taken using bandpass filter of 610–630 nm.

For analysis of cell permeabilization the propidium iodide (PI) (ThermoFisher Scientific Inc., USA) fluorescence dye was used. Just before electroporation, 63 μl of cell suspension were mixed with 7 μl of 300 μM PI to obtain final dye concentration of 30 μM. For PEF permeabilization, the 60 μl of the resultant suspension was transferred to 1 mm gap electroporation cuvette (Biorad, Hercules, USA). After electroporation, the cells were instantly transferred to 1.5 mL tubes (Eppendorf, Hamburg, Germany) and incubated for additional 10 min at room temperature followed by flow cytometric analysis (Amnis, Seattle, USA). The fluorescent cells (PI permeable) were gated as permeabilized in accordance with standard gating strategy used in electroporation studies^55–57^. After electroporation, a shift of spectra has been observed and the cells in the gate (which was defined based on the untreated control) have been interpreted as fluorescent positive (permeabilized), while the cells outside the gate as fluorescent negative (non-permeabilized). The number of the PI fluorescent cells in the untreated control did not exceed 10%. The darkfield (side scatter), brightfield and fluorescence images of each cell (total of 10000 cells for each unique set of parameters) were taken. At least three independent repetitions were performed. The examples of acquired fluorescence images are shown in Fig. 7.

Only single cells were used in analysis. The permeabilization rates based on visual confirmation of PI electrotransfer in each sample were in agreement with percentages acquired after the definition of the gates.

### *In vivo* model

In this study BALB/c mice were used. For experiments, the flanks of the animals were shaved, depilated using 8% Na_2_S × H_2_O and then rinsed with water. Subsequently, suspension of *P*. *aeruginosa* in PBS (OD = 8 at 600 nm), was inoculated onto the surface of skin using cotton swab. One hour later the mice were anesthetized by intraperitoneally injection of ketamine (80 mg/kg) and xylazine (10 mg/kg). After anesthesia was established, the mice were imaged with IVIS Spectrum (Caliper Life Sciences) using Living Image Software to confirm bacterial contamination. The animals with bacterial contamination were treated with 25 kV/cm 900 ns pulses (a total of 1000 pulses) at repetition frequency of 15 kHz. 150 µl of distilled water, 0.1% or 1% acetic acid was used as a skin-electrode interface. The “tweezer” type electrodes were applied (gap 1.2 mm) and a total of 70 ± 5 pulsing sequences were delivered to each mouse by accurately repositioning the electrodes to cover the whole treatment area. As a result, the treatment took less than 2–3 min and a total of 70000 ± 5000 pulses were delivered. The deviation in the pulsing number was influenced by the human factor since it was not always possible to accurately reposition the electrodes, therefore some overlapping occurred and more electrode repositions were required to cover the area. After the treatment mice were again analyzed with IVIS Spectrum.

The protocol was approved by the State Food and Veterinary Service (license Nr. G2–48) and the study was carried out in strict accordance with the recommendations in the Guide for the Care and Use of Laboratory Animals. All applicable international, national and/or institutional ethical guidelines were followed.

### Spectrophotometry

The luminescence of bacteria was evaluated using a Synergy 2 microplate reader and Gen5^TM^ software (BioTek, USA). After electroporation the samples (120 μl) were distributed in to the wells of white 96-well flatbotom plate corresponding to different set of parameters. The luminescent signal was measured kinetically for a period of 10 h with 2.5 min intervals. The luminescence read time was 2 seconds. Beforehand, the changes in RLU (relative luminescence units) were compared to conventional clonogenic assay^13^ using several random data points and it was confirmed that bioluminescence assay is accurate to represent viability, i.e. CFU (colony forming units). The values of RLU were in good agreement with CFU with maximum deviation in the range of 10–15%.

### Statistical analysis

One-way analysis of variance (ANOVA; P < 0.05) was used to compare different *in vitro* treatments. Tukey HSD multiple comparison test for evaluation of the difference was used when ANOVA indicated a statistically significant result (P < 0.05 was considered statistically significant). All experiments have been performed at least in triplicate and the treatment efficiency was expressed as mean ± standard deviation. For *in vivo* data, the Mann Whitney test was used. All data were post-processed in OriginPro software (OriginLab, Northhampton, MA, USA).

## Data Availability

Derived data supporting the findings of this study are available from the corresponding author V.N. on request.
